# Experiences and reflections of Somali unaccompanied girls on their first years in Sweden: a follow-up study after two decades

**DOI:** 10.1108/IJMHSC-03-2018-0018

**Published:** 2018-09-10

**Authors:** Magdalena Bjerneld, Nima Ismail, Soorej Jose Puthoopparambil

**Affiliations:** Department of Women’s and Children’s Health, Uppsala University, Uppsala, Sweden

**Keywords:** Sweden, Support, Somalia, Girls, Unaccompanied children

## Abstract

**Purpose:**

Unaccompanied asylum-seeking children (UASC) from Somalia are one of the largest groups of UASC in Europe and Sweden. The current study is a follow-up of a Swedish study conducted in 1999, where unaccompanied asylum-seeking girls (UASG) from Somalia were interviewed. In 2013, UASG from the 1999 study were interviewed again, as adults who have settled and found a new life in Sweden. The purpose of this paper is to explore how these women experienced their transition into the Swedish society.

**Design/methodology/approach:**

A qualitative research design using semi-structured interviews was adopted for this descriptive study. Thematic analysis was used to analyze the data.

**Findings:**

UASG need support from different groups of adults, ranging from the staff at the group homes to community members, including countrymen, to establish a good life in their new country. The UASG need understanding and knowledgeable staff that can support them through the initial period, when they do not have their parents close to them. All actors in the supporter network need more knowledge about the difficulties in war situations. Former UASC can assist newcomers as well as being informants to authorities in a new country. Both parties involved need to be open and willing to learn from each other.

**Research limitations/implications:**

UASG who consider themselves successful in being integrated into the Swedish society were interviewed and, therefore, the study mainly describes aspects that promote integration.

**Originality/value:**

There are limited follow-up studies on how UASG have experienced their life after almost two decades in the new country.

## Introduction

The United Nations High Commissioner for Refugees (UNHCR, 1994) defines an unaccompanied child as:

[…] a person who is under the age of eighteen, unless, under the law applicable to the child, majority is, attained earlier and who is separated from both parents and is not being cared for by an adult who by law or custom has responsibility to do so.

Sweden has a long experience of receiving unaccompanied children. The most well-known groups are Jewish children from Germany during the 1930s, Finnish children during the Second World War (Eriksson, 2013), children from Hungary during the crisis in 1956 (Hessle, 2009), and children from Chile and the Middle East during the 1970s (Angel and Hjern, 2004). In the early 1990s, unaccompanied asylum-seeking children (UASC) from Somalia were the largest group of UASC in Sweden and this continues to be one of the largest groups to date (Swedish Migration Agency (SMA), 2017a, b). A record number of unaccompanied children sought asylum in the world in 2015 (UNHCR, 2016), of whom about 35,400 came to Sweden. In 2016, the number decreased to 28,939 children, and in 2017 the number was 1,336 UASC (Swedish Migration Agency, 2018).

Until 2006, the Swedish Migration Agency (SMA) was responsible for processing the asylum applications and for providing accommodation for the UASC (Swedish Government, 2005). However, Save the Children and other NGOs have reported that many children did not receive the psychosocial support they needed, as the system made it difficult for the SMA staff to both represent the authority and to create supportive and trustful relationships with the children (Brendler-Lindqvist, 2004). Since 2006, the municipalities where the children are placed by the SMA have been responsible for arranging the accommodation, schooling, and other aspects of their daily lives (SMA, 2017a, b). Children under the age of 14 years are often placed in family homes; either with relatives, or with Swedish families. Older children live in group homes run by the municipality or by the private sector (SMA, 2017a, b; Fälldin and Strand, 2010).

However, there are many different organizations, such as the SMA, municipalities, the county council, and the school system, which provide the UASC with care and support in Sweden. A report by The National Board of Health and Welfare concerning healthcare and schooling for children living in group homes highlighted the risk of no one organization taking overall responsibility for the children due to lack of coordination. The report indicated that if the physical and psychosocial abilities of the child are assessed and the support is coordinated, the possibility for the child to succeed in school is higher (The National Board of Health and Welfare, 2013).

Although unaccompanied boys are a larger group among UASC, unaccompanied girls have come to western countries such as Sweden. These girls have often been exposed to the risk of being sexually abused during conflicts in their home countries or during the flight (Human Rights Watch, 2016; Derluyn and Broekaert, 2007). The projection for the coming years indicates that about 20 percent of the UASC coming to Sweden will be girls (SMA, 2017a, b). Girls’ needs might be different to those of boys because of factors such as different gender roles in the home and host countries (Keles *et al.*, 2016), increased vulnerability (Birchall, 2016) and difficulties in living in group homes together with boys (Kaukko and Wernesjö, 2016).

Somalia is one of the poorest countries in the world with a long experience of war. The majority of the population live in a pastoral, patriarchal society (UNDP, 2012). Traditional gender dynamics are the norm, where husbands make most decisions and have far greater power than wives (Connor *et al.*, 2016 ). In this study, we describe the process through which Somali women, who arrived in Sweden as UASC from Somalia during the 1990s and early 2000s, found their way into society. The support these women experienced during this process is the focus of this paper.

### Support for UASC

Each child has different needs depending on previous experiences and current circumstances (Hopkins and Hill, 2010). UASC are under enormous stress during their migration after having left their parents. Often, they have experienced traumatic events in the home country, and in the host country they do not know the language, the culture, and they do not know whether they will receive permission to stay (Brendler-Lindqvist, 2004; Keles *et al.*, 2016). As children, they are highly dependent on support from the community in which they live to assist them with the practical matters, emotional support, and building new relationships that can create a feeling of belonging (Kovacev and Shute, 2004; Wernesjö, 2012).

Separation from both parents during adolescence is linked to a higher risk of mental health problems, such as anxiety and posttraumatic stress symptoms, including depression among children, especially for girls (Derluyn *et al.*, 2009; Derluyn and Broekaert, 2007). However, the stress the children feel upon arrival can be buffered by the support provided by the host community, including where staff provide a variety of services (Mels *et al.*, 2008).

A study in Norway reported that UASC from Somalia, especially girls, have lower levels of depression compared to other UASC (Seglem *et al.*, 2011). However, the research, so far, has not been able to fully explain this result. It is possible that the extent to which the girls identify with the Somali culture and show they are Muslims (wearing a headscarf) plays an important role. Somali girls, in a study in the USA, who upheld their identity as Somalis had better mental health than those who dressed and behaved like the host population (Ellis *et al.*, 2010 ).

Earlier research has indicated that children’s resilience, defined as the “capacity to do well despite adverse experience” (Gilligan, 2000), can be important for the children’s well-being during their first years in the new country. Other protective factors, such as personal strength, a stable family in the home country, and strong supporting values and norms in the host country, are also important for their integration (Fazel and Stein, 2002).

The most common group providing support to UASC is social workers. Based on interviews with social workers in the UK, Kohli has described how they give UASC support in three different ways. The first domain he calls “cohesion,” which refers to “bringing order to the outside world,” where the social workers meet the practical needs and work in the perspective of the “here and now” to establish trust in the children. A staff member who works in this way is realistic and pragmatic and acts as advocate, protector and mentor.

The second domain is called “connection,” which relates to “making peace in inner worlds,” where the social worker listens to the children’s stories from the flight and to their feelings, trying to allow them to experience a feeling of control and be of being prepared to plan for the future. Kohli suggests this support represents the missing parents.

In the third domain, called “coherence,” the social worker is one who supports the children in organizing their daily work, acts as a “parent *in situ*” and ensures the best desired outcome for the child. This person often stays with the child for a long period of time and builds up the trust between the child and the staff and, therefore, can contribute to making long-term plans (Kohli, 2007). These concepts, as described by Kohli, can assist in understanding the different kinds of support that are needed for the well-being and integration of unaccompanied children.

### The study in 1999

In 1998–1999, a group of 14 UASC (girls) from Somalia was interviewed about their experiences during their very first years in Sweden. They were between 10 and 17 years of age when they arrived in Sweden and had, on average, been in the country for four years. The girls were recruited through a modified snowball sampling technique (Dahlgren *et al.*, 2007) where we contacted adult Somalis, school nurses, program officers in communities, and heads of group homes. These contacts led us to some of the girls, who in turn recommended friends, who were recruited to the study. The first author interviewed the girls in Swedish or with the second author, a Somali Medical Doctor settled in Sweden, interpreting. The second author also acted as a cultural Broker, who provided contextual understanding to the first author (Tribe and Raval, 2002). The interviews were audio-recorded and lasted for about 60 minutes each and took place in a location chosen by the girls, for example, in their schools or their group homes.

In summary, the girls had left Somalia because, during the war, there was a high risk of them being raped. They found their travel to Sweden frightening because they did not know where they were going and still were afraid of being raped. They considered the reception system in Sweden to be good, but they lacked others to talk with and longed for their mothers. The girls thought that their integration into the Swedish society so far had gone well. The main difficulties were the language and the differences between the Somali and the Swedish cultures, including food habits and gender norms (Bjerneld, 1999).

The current study is a follow-up of the study conducted in 1999. In 2013, there was an opportunity to interview some of the participants from the previous study, who were now adults, and who had settled and had found a new life in Sweden. The aim of the present study was to explore how a group of women who arrived as unaccompanied girls from Somalia experienced their transition into the Swedish society. A qualitative research design using semi-structured interviews was adopted for the descriptive study.

## Methodology

### Study participants

Of the 14 women who were interviewed in the 1998–1999 study, we were able to find eight, each of whom were invited to participate in the current study. Five of them participated. Of the three who declined, one was sick, another had moved out of the country, and the third declined the invitation without providing any explanation. When we searched for the previous participants, we met seven other Somali women who had come to Sweden as UASC girls during the 1990s and early 2000s. To enrich our data, we decided to include these women in the present study. The inclusion criteria were: arrived in Sweden as an UASC, had been in Sweden for at least five years, and had obtained permanent residency (PR) in Sweden. When we interviewed the girls in the first study, not all of them had obtained PR. We found the ones without PR to be extremely worried about their future in Sweden and had difficulties reflecting on their experiences and focusing on the interview. Therefore, we decided to retain the PR status as one of the inclusion criteria in the current study. Table I presents the demographic characteristics of the women. The first five women were part of both the 1999 study and the current study.

### Ethical considerations

Ethical approval for the study was obtained from the Regional Ethical Review Board, Uppsala, Sweden. The participants were provided with information regarding the aim of the study, the voluntary nature of their participation, the confidentiality of the information obtained from the study, and the contact details of the first author in both verbal and written (information sheet) formats. Written informed consent was obtained from all participants.

### Data collection

Inspired by the results of the study in 1999 (Bjerneld, 1999) and other studies on UASC (Kohli, 2007; Kohli, 2011; Mels *et al.*, 2008; Wallin and Ahlström, 2005), an interview guide exploring the women’s experiences of the healthcare system, their schools, and other services, was developed. In the previous research on UASC, there is very little information about the family situations in the home countries (Kohli, 2007; Eide and Broch, 2010). Most researchers start to ask about children’s experiences from the moment they enter a European country (Kohli, 2011). We, therefore, asked the women about what they remember from their childhood in Somalia and how their background might have influenced their life in Sweden.

In the current study, interviews were performed by the first author in Swedish. The second author was present during six of them. The atmosphere during the interviews was relaxed and the women talked freely about their experiences and there were no time restrictions on the interview. The interviews were audio-recorded and lasted for about 60 minutes each and took place in a location the women chose, for example, coffee shops, or their homes. Finally, the interviews were transcribed verbatim. No major differences were observed between the interviews where both the authors were present and only one of the authors was present.

### Data analysis

We analyzed all the transcripts using thematic analysis (Braun and Clarke, 2006). The first author did line-by-line coding and later collated all related codes to sub-themes. They were further organized to develop themes. The first and last authors reviewed and revised the sub-themes and themes, resulting in four themes describing the experiences of the interviewees. Second, wherever applicable, we compared the results of the 1999 study with the present study.

## Results

The 12 women had been in Sweden on an average of 17 years. They considered themselves to be integrated into the Swedish society and to have a good life. We found the two groups, women who participated in both studies and the women who participated in the current study alone, told us very similar stories regarding their experiences in Sweden. We use the term “girls” to refer to the participants in the 1999 study and “women” to refer to the participants in the current study. The factors that helped the women to integrate or adapt in Sweden are described in two themes: “Stable family support and motivation” and “Supportive persons to talk to.” The factors that they found challenging are described in the two themes: “Lack of understanding of girls’/women’s previous lives,” and “Being a female.” The numbers after the citations reflect the participant numbers indicated in Table I.

### Stable family support

In the first set of interviews, the girls talked a lot about the war and the family they had left and how they missed their mothers. The girls came from an urban, patriarchal society, and from middle-class families where their parents had their own businesses or were academics. The mothers had the dual task of caring for children at home and conducting their businesses in the city. Both the girls and the women described their youth as a time when they felt safe and lived in a stable family with affectionate parents, which made them feel mentally strong and secure. According to the women, what gave them the confidence to manage their new life in Sweden was their childhood, illustrated below:

We had a good childhood. We had loving parents. We received a stable foundation and it was very good. I think that has helped us very much

(1, 2013).

The stable family situation became unstable during the war. A common reason for the participants to leave Somalia was that the fathers were killed by the militia and the mothers and relatives could not manage the situation. There was a high risk for the young girls to be raped or forcibly married to one of the men in the militia. One of the women had witnessed her father being murdered by the militia and consequently the mother thought it was too dangerous for her daughters to stay in Somalia and decided to send one of them to Europe. In both the 1999 study and in the current study, the women talked about how difficult it had been to leave their mothers in Somalia, who they perceived as being “strong” and self-reliant, and they, therefore, became their role models.

The mothers had been their most important supporter in life, and, in Sweden, they felt lonely, they missed their mothers and felt they had no one to talk with. One of the girls said that she cried all the time and dreamt about the mother and another said she wrote poems to her mother every day as a way of coping.

### Supportive persons to talk to

In the first study, the girls not only talked about how they missed their mothers, but, as mentioned earlier, they also spoke about the fact they had no one with whom they could trust and talk to. However, one of the participants had a good friend who she could call and talk to, and another became acquainted with a Swedish family to whom she could go whenever she wanted. She described them in the following words:

It feels I am part of the family […] We have close contact […] I can go to them whenever I do not want to be alone or to get help with my homework or have dinner, I am always welcome

(4, 1999).

Although the women still missed their mothers, they talked about how they had met compassionate individuals in Sweden, who encouraged, supported, and cared for them and became their new role model. These individuals were staff at their group homes, teachers, foster parents, social workers, or bosses at their workplace. They saw each girl’s potential and the women considered these persons to be the most important factor that helped them to be integrated into the Swedish society. They were trustful, showed patience and empathy, and had boosted their self-confidence so they became strong, for example, to continue at school. One woman described her supportive person in this way:

She was like an extra mum, encouraged us all the time, and believed we would manage. She was an adult who understood me, my world. She played a very important role in my life […] One needs someone to look up to […] It is difficult to describe her but, she is wonderful. She was there for everyone, not only for me […] Once I told her, I wished she was my mother and she gave me a hug

(4, 2013).

Some of the women were traumatized by the war and needed to talk to someone about their experiences. However, it was not easy to talk about this, even with professionals. One girl had a bad experience at a psychiatric clinic for young people. She had met many different counselors during the first year and felt they only repeated the same question about why she had come to Sweden, instead of helping her by asking further questions. Another woman said it was a question of trust. She considered herself lucky to have met a midwife at the clinic for young people with whom she felt she could talk about everything and who she trusted, because she was more like a friend than a professional counselor.

In the 1999 study, the girls told us that sometimes they had difficulties concentrating in school because they were very worried about their future and their family in Somalia. However, their mothers had emphasized how important it was to educate oneself to get a job and support oneself, which motivated them to continue. One woman remembered her difficulties during the initial years in school and had, therefore, worked as an extra teacher when ordinary teachers did not feel they could understand and help the Somali children. She told a story about a boy, an UASC, who was worried about his relatives in the home country. The worry hindered the learning:

He did not want to go to school. When I talked to him it was different […] He was more open when I was there and returned to school some days after we had met. Language, culture, competence, a lot of things influence. I can say I have also been in the same situation and I have succeeded

(4, 2013).

### Lack of understanding of the girls’/women’s previous lives

Groups who had a responsibility to support the girls were the staff at the group homes, teachers in schools, and healthcare staff. Although most of the participants considered the staff to be very engaged and kind, they often concluded that the staff lacked an understanding of life in Somalia, which created challenges during their early years in Sweden. One of the participants remembered how she was given a map and asked to go to the SMA office. She had never used a map before and did not know how to travel using public transport. She ended up not finding her way to the SMA office.

Some of the women remembered how they, when they first arrived, were very thin due to lack of sufficient food during the war, and in some cases due to the long journey to Sweden. This influenced their physical and psychological health. Some were sick and depressed when they arrived in Sweden and, therefore, had difficulties eating regularly. One participant said that she felt that the staff at the group homes did not understand why she felt sick and depressed, which made her condition worse:

I wanted to eat, but I could not. I had not eaten real food in many years and now I was supposed to eat regularly. It was difficult. I was very worried […]

(1, 2013).

Another participant faced difficulties in eating during the initial period because she was not used to refrigerators. She presumed that the same salad that had been served two days in a row was old and not edible.

Generally, the women had a positive experience of the Swedish healthcare system. Most of the staff they met were committed and dedicated. However, the women’s lack of understanding of the Swedish healthcare system and the healthcare professionals’ lack of understanding of life in Somalia was a challenge. During their time in Somalia, maternal healthcare did not exist. Therefore, the women said they did not understand the importance of antenatal visits and did not initially attend them. When they arrived in the delivery clinic, not all of them were treated well by the healthcare staff. One woman had met a midwife who did not understand that she was circumcised. She thought the woman had a burn. The woman had to use a lot of energy during her labor to explain this to the midwife.

Some of the women told stories about how they felt that they were being questioned by healthcare staff, who had negative preconceptions about how immigrants exaggerated their symptoms. One woman, instead of receiving information and support, had an upsetting experience when the doctor did not examine her child who was very sick with pneumonia:

My baby boy got pneumonia. When I went to the health centre, they said it was a common cold and the doctor told me to return home […] The next day he became worse […] I phoned to another health centre [in a community where few immigrants live] […] They immediately referred us to the hospital

(4, 2013).

Although the participants considered themselves integrated, their experiences show that integration is still an ongoing process for them. The women said they had both Swedish and Somali friends, but one of the participants expressed a need to have more contact with Swedish families to learn more about each other’s way of life. However, she thought it was difficult to get in touch and socialize with Swedish parents. One woman said she often met Swedish parents at her children’s pre-school and both parties considered it a good idea to meet and socialize. However, this never happened, and she attributed it to the busy schedule of the Swedish parents.

### Being a female

The girls in the 1999 study talked about the difference between how boys and girls were supposed to behave in Sweden and Somalia. They talked, for example, about the use of alcohol and having sex before marriage. In the first set of interviews, the girls were already talking about how they appreciated the freedom in Sweden, the absence of war, the opportunities to study, and the fact that everyone was equal. In the group homes, boys and girls lived together peacefully.

However, during the early years in Sweden the girls experienced clashes between how the Somali culture and Swedish culture perceived young women. One woman remembered how challenging it was for her to move from the group home to an apartment when she turned 18 years old. In Somalia, it would be unrealistic to let a girl live alone in an apartment, and, therefore, other Somali people in Sweden made comments about this, insinuating that the girl was promiscuous. Hence, she and her other female friends found it very difficult to leave the group homes and live alone in an apartment. They felt very lonely in this situation.

In the current study, the women still talked about the freedom of women, but they also spoke about gender roles. During the initial years in Sweden, one woman thought there was something wrong with the Swedish women, as they did not provide a full service to their husbands, which to them signified taking care of all housework and the children. But, over time, she understood that equality in housework chores is important. One woman said that her husband was even questioned by other Somali men, when he helped her to take care of their children. She said:

Many Somali people think my husband is a strange man since he can be at home changing diapers when I attend a meeting

(1, 2013).

Finally, we asked both the girls and the women whether they thought there was a difference between being either an unaccompanied girl or boy when arriving and living in a country such as Sweden. Some thought it was up to the individual. Some of the women gave us examples of boys who had managed very well in Sweden. Others thought girls, in their home countries, learned how to take care of themselves and family members, meanwhile boys most often receive this service from others. One woman described the expectations when they lived in Somalia in this way:

The girl should manage very well in school. When she comes home from school she takes care of the home. The boys are also supposed to manage well in school but, when they return home from school the women have prepared everything for him

(11, 2013).

In 1999, the girls dreamt of getting married and having children, to educate themselves to join a profession where they could help others or work internationally, such as doctors, nurses, or pilots. All participants, but one, in the current study were married and had children. They appreciated the opportunities they had in Sweden to study, to get a job and to be able to support themselves. The women had professional roles, as indicated in Table I, but wanted to further to educate themselves when their children are older. They said they wanted to have a career as the Swedish women did. One wanted to be a home language teacher, another a midwife, and a third, a politician. They saw it as a continuation of the integration process, but also a way to repay the Swedish society. The women indicated that learning and improving their ability to use the Swedish language was still a challenge and considered it easier to learn as a child than as an adult.

## Discussion

This qualitative study describes the experiences of 12 women from their arrival as UASC from Somalia to establishing themselves in Sweden. They had been living in Sweden for almost two decades, which has given them the opportunity to reflect upon the process of being part of the Swedish society.

Comparing our results to those of prior studies that included both boys and girls (Thomas *et al.*, 2004; Hopkins and Hill, 2008), the reasons for their flight were similar in the sense that girls also fled due to war and conflict, persecution of family members, and the risk of rape in their home country. They did not tell us that they had been exposed to any sexual harassment or rape, either because it had not happened or because they did not want to talk about it; we do not know.

The women in the present study talked about the fact that they were prepared from childhood to take care of themselves and others. This might mislead us to suggest that unaccompanied girls from Somalia are better prepared than boys to take care of themselves in a new society. In the practical part of life, such as doing housework, girls probably are well prepared, but still, they need emotional support, just as boys do, or more due to the risk of being exposed to sexual violence. However, further research is required to better understand the varying needs of boys and girls and the varying gender roles in their countries of origin.

### Need for support from different groups in society

Our results indicate that the participants considered themselves to be well-motivated to succeed in their new country. They still needed different kinds of support from different kinds of professionals, but this support had to be obtained from their own ethnic group as well as from the public. The participants described their need for practical support in the form of basic needs such as housing, food, schooling, etc. They also talked about how they had needed someone to talk to and someone who could teach them about what daily life in Sweden is like. Therefore, we chose the concept developed by Kohli (2007) to describe the three different kinds of support that UASC need for their well-being and integration into a host country. In the current study, we explored the support that our participants received from all actors, not only that provided by social workers. In addition to the three dimensions of support described by Kohli, we also identified the dimension of companionship, a type of support not expected from professionals such as social workers but described as being important for promoting the feeling of being integrated.

### Cohesion, connection, coherence, and companionship

Trust in staff and other adults, safety, being accepted by society (Eriksson, 2013; Kohli, 2007), and having a feeling of being welcomed and loved are important factors for the well-being of migrant children (Hjern, 2006). The staff at the group homes and the adults in the family homes have a duty to assist the children with practical support (cohesion). The participants described that they appreciated the staff, but sometimes the staff lacked understanding of the UASC’s country of origin. One of the women illustrated this with her story about how she had difficulties finding the SMA office, as she nor her friends knew how to use public transport or how to read a map. This demonstrates how cohesion could be improved by training the staff and improving their knowledge of the daily lives of the UASC’s country of origin, where amenities that are often considered basic or inevitable in developed countries might be unavailable.

UASC who suffer from psychological difficulties and worries about their asylum process need support from the staff to cope with their daily lives (Malmsten, 2014; Godani, 2016). The participants stressed the importance of emotional support, or what Kohli (2007) describes as connection, which could help them to cope with stressful situations. They indicated that listening and comforting are the most important skills required for staff working with UASC, which is vital for UASC’s well-being. When they were girls, they said they needed someone to talk to about their memories of the war, their flight, and the longing for their mothers. In Somalia, this role was fulfilled by their mothers, which made the girls feel secure and gave them good self-confidence. If the staff in the group homes functioned well, they could give the children guidance about to how to cope with difficult feelings, and the participants described these individuals as “an extra mum.” Previous studies have described the importance of such peers being kind, friendly, and open (Newbigging and Thomas, 2011; Gilligan, 2000). As highlighted in the results, the midwife who adopted a friendlier approach was more successful in communicating than the “trained” psychologist. This exemplifies the professionals’ challenges in knowing how to talk with children from different countries, who might have been through adverse situations, and how to make them feel safe and supported despite the worries in these children’s daily lives (Godani, 2016; Malmsten, 2014).

The third domain described by Kohli (2007) is coherence: “resettlement as regenerating the rhythm of ordinary life,” where the UASC build a new life with the assistance of social workers which provides a long-term perspective. One of the women talked about her previous lack of knowledge of the importance of visiting an antenatal health clinic, which indicates that there is a continued need for guidance in girls’ integration into the host country, not only during the initial phase when the UASC are living in the care of public service, for example, in group homes, but also during later stages.

A fourth dimension of support, not described by Kohli is companionship that was illustrated from two perspectives in the present study; close contacts to same ethnic group and contacts with the host population. Coherence, as described by Kohli, requires the person who provides the support to act as a “parent *in situ*” and often stays with the child for a long period of time. The individual offering companionship is not required to satisfy the above-mentioned conditions. They act as a companion to whom the unaccompanied individuals, as a child or adult (at a later stage), can turn to get support and have a normal life. Moreover, this is not expected to be a paid task carried out by NGOs or social workers. This type of support must be developed by the adults in cooperation with the UASC. The woman who worked as a language teacher was one example of contact with the same ethnic group, as she could support an UASC in a special way, because she could understand the child and give hope for the future. This was not her main task, but, as an adult originating from the same country, she could be a role model for the girl, helping her to think about what she could achieve in the future. In Sweden, two different follow-up studies with UASC from different countries, ten years after their arrival, reported that the children had succeeded in establishing themselves in Sweden, despite the fact that they had few contacts with the host population (Wallin and Ahlström, 2005), but they had received valuable support from the same ethnic group living in Sweden (Hessle, 2009) and a study in Belgium with unaccompanied boys reported that the ethnic community had an important avoidant/distractive companionship role, enabling the boys cope with stress (Mels *et al.*, 2008). However, our participants also highlighted the other side of the coin in being in close contact with people from the same ethnic group was understood to decelerate their learning of Swedish.

The participant in the present study considered it important for UASC to mingle with the locals to improve their language skills and learn about the culture. One of the girls talked about how important her Swedish contact family had been for her well-being during her first years in Sweden. However, one approach to companionship cannot be considered better than the other. The best approach might be a mix of both, where the children can meet and receive support from individuals from their home country while learning about the new country and culture by meeting other groups from the new home country. Documentation of support from the host population is limited and further research would inform us whether it is the ethnic background, the experience of being UASC, or whether it is the character of the persons that is important in the dimension of companionship.

### Staff training and supervision

The women in our study stressed the importance of recruiting staff who are interested in learning about the children’s home country, those who are positive, supportive, and trustful, and who can listen to children’s, often traumatic, stories many times and still be empathetic. To understand the children, staff needs to have knowledge about unaccompanied children’s specific needs and vulnerability. If they are not trained sufficiently, they can encounter problems knowing how to communicate with UASC (Godani, 2016). The example of the midwife having better skills in how to communicate than the psychologist is one example of this. Social workers often avoid asking questions about refugee children’s background in order to avoid their own difficulties in managing emotional distress or because they are afraid to cause any emotional distress for the child (Eriksson, 2013; Godani, 2016), which highlights not only the need for training but also support, as well as effective supervision of staff. Earlier studies have suggested that staff training should include special health needs, such as normal reactions to traumatic experiences, an understanding of the culture in the children’s countries of origin, intercultural communication practice (Human Rights Watch, 2016; Newbigging and Thomas, 2011), and psychosocial support (Mels *et al.*, 2008). Additionally, we suggest that discussing the different gender roles in different parts of the world will increase the staff’s understanding of what they can expect from boys and girls, respectively, and how their roles differ in the various countries of origin.

The support framework provided by Kohli could be a valuable tool in developing staff training modules. Based on our participants’ experiences, the staff and other professionals working with UASC need training in addressing the cohesion and connection aspects of the support. It may not be possible to address coherence, the long-term aspect, through such training. Moreover, it might not be feasible to expect the staff to provide this long-term support. As described by our participants, this support must come from the host society, and must include both Swedish people as well as other migrants from the same country of origin.

## Conclusions

Sweden has a long history of receiving and providing support to UASC. Today, Sweden must care for more UASC than ever, including an increasing number of UASG.

The study highlights the UASG’s need for support from different groups of adults, ranging from the staff at the group homes to community members, to start a new life in their new country. The different supporters replace the mothers or other individuals, who are their role models, but with whom the UASG have lost contact. The UASG need understanding and knowledgeable staff that can support them through the initial period, when they do not have their parents and friends close to them. The results are relevant for the larger UASC group. They also need countrymen who can guide them and give them a long-term perspective of their new home country.

All actors in the supporter network need more knowledge about the difficulties in war situations. Former UASC can assist newcomers as well as being informants to authorities in a new country. Overall, both parties involved, the UASC and the supporting actors in the host country, need to be open and willing to learn from each other.

## Figures and Tables

**Table I:** General demographic characteristics of the participants in 2013

	Age when left Somalia	Age when interviewed	Years of residence in Sweden	Education/profession	Professional status
1	17	34	17	Social worker	Employed
2	16	37	21	Social worker	Employed
3	14	35	21	Care worker	Employed
4	14	33	19	Economist	Employed
5	11	30	19	Student (nursing)	Student
6	14	24	10	Pre-school teacher, home language teacher	Employed
7	13	29	16	Nurse	Employed
8	17	42	23^a^	Nurse/medical student	Student
9	17	23	6	Student (economics)	Student
10	17	30	13	Student (social work)	Student
11	14	30	16	Pre-school teacher	Employed
12	17	38	21	Nurse tutor, doctoral student	Employed

**Note:** ^a^Fled to another country before she arrived in Sweden
